# Assessing Adverse Health Effects of Long-Term Exposure to Low Levels of Ambient Air Pollution: The HEI Experience and What’s Next?

**DOI:** 10.1021/acs.est.3c09745

**Published:** 2024-07-11

**Authors:** Hanna Boogaard, Dan L. Crouse, Eva Tanner, Ellen Mantus, Annemoon M. van Erp, Sverre Vedal, Jonathan Samet

**Affiliations:** † Health Effects Institute, 75 Federal Street, Boston, Massachusetts 02110-1940, United States; ‡ Department of Environmental & Occupational Health Sciences, University of Washington, 4225 Roosevelt Way N.E., Seattle, Washington 98105, United States; § Department of Environmental & Occupational Health, Department of Epidemiology, Colorado School of Public Health, 13001 East 17th Place, Aurora, Colorado 80045, United States

**Keywords:** air pollution, long-term exposure, mortality, epidemiology, policy

## Abstract

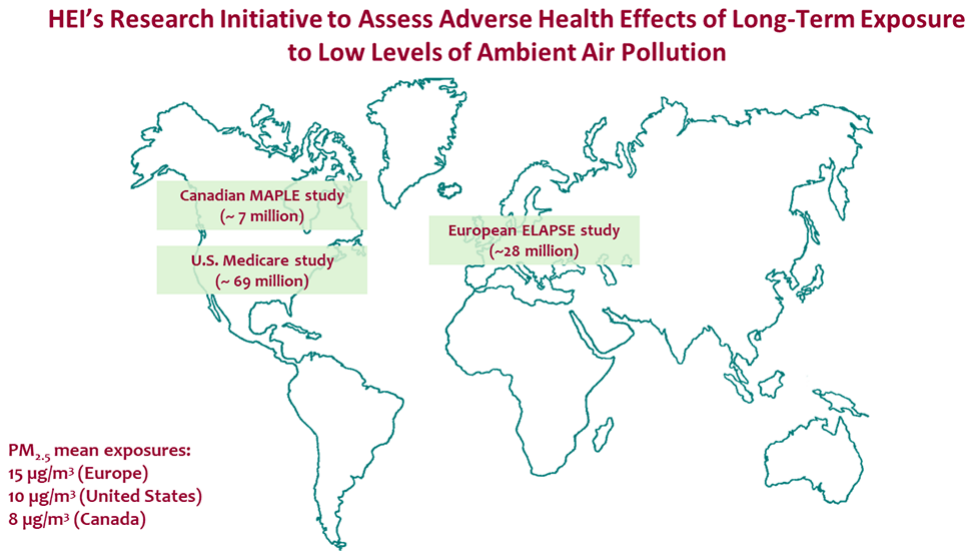

Although concentrations of ambient air pollution continue to decline in high-income regions, epidemiological studies document adverse health effects at levels below current standards in many countries. The Health Effects Institute (HEI) recently completed a comprehensive research initiative to investigate the health effects of long-term exposure to low levels of air pollution in the United States (U.S.), Canada, and Europe. We provide an overview and synthesis of the results of this initiative along with other key research, the strengths and limitations of the research, and remaining research needs. The three studies funded through the HEI initiative estimated the effects of long-term ambient exposure to fine particulate matter (PM_2.5_), nitrogen dioxide, ozone, and other pollutants on a broad range of health outcomes, including cause-specific mortality and cardiovascular and respiratory morbidity. To ensure high quality research and comparability across studies, HEI worked actively with the study teams and engaged independent expert panels for project oversight and review. All three studies documented positive associations between mortality and exposure to PM_2.5_ below the U.S. National Ambient Air Quality Standards and current and proposed European Union limit values. Furthermore, the studies observed nonthreshold linear (U.S.), or supra-linear (Canada and Europe) exposure-response functions for PM_2.5_ and mortality. Heterogeneity was found in both the magnitude and shape of this association within and across studies. Strengths of the studies included the large populations (7–69 million), state-of-the-art exposure assessment methods, and thorough statistical analyses that applied novel methods. Future work is needed to better understand potential sources of heterogeneity in the findings across studies and regions. Other areas of future work include the changing and evolving nature of PM components and sources, including wildfires, and the role of indoor environments. This research initiative provided important new evidence of the adverse effects of long-term exposures to low levels of air pollution at and below current standards, suggesting that further reductions could yield larger benefits than previously anticipated.

## 1 Introduction

Ambient air pollution is a major contributor to premature mortality and morbidity worldwide. There is now an evidence-based broad consensus that exposure to air pollution causes an array of adverse health effects; the supporting literature has grown exponentially since the mid-1990s.^1−5^ Air pollution damages most organ systems and is linked to many debilitating diseases, such as asthma, cardiovascular diseases, chronic obstructive pulmonary disease, pneumonia, stroke, diabetes, lung cancer, and dementia.^6^ Estimates from the Global Burden of Disease (GBD) 2019 study ranked air pollution as the fourth global risk factor for premature mortality, surpassed only by high blood pressure, tobacco use, and poor diet.^7,8^

Air pollution concentrations have been declining over the past few decades in many high-income countries, due largely to successful air quality regulation and subsequent reductions in emissions from major air pollution sources, including transportation and power generation. Nonetheless, recent studies have reported associations with health effects in the general population at levels even below current ambient air quality standards.^9−11^ On the basis of this mounting evidence, the World Health Organization (WHO) released new Air Quality Guidelines (AQG) in 2021. They recommended that annual mean concentrations of fine particulate matter (PM_2.5_) and nitrogen dioxide (NO_2_) should not exceed 5 and 10 μg/m^3^, respectively, noting that adverse health effects were documented to occur above these values.^5^ However, few studies have described in detail the shape and magnitude of the risk relationship between health outcomes and exposure to pollutants at the low end of the global exposure range; the exposure-response function (ERF). ERFs are used to quantify the health effects of past exposure to air pollution for policy purposes and to predict the health benefits of future reductions in air pollution.

To address this critical gap, the Health Effects Institute (HEI) has completed a comprehensive research initiative to investigate the health effects of long-term exposure to low levels of air pollution in the United States (U.S.), Canada, and Europe. The initiative was motivated by reports of adverse effects in the range of national air quality standards and the consequent need for more certain evidence at these risks, both to confirm the finding of adverse effects and to develop ERFs in support of regulatory decision-making. Low levels of air pollution were defined as levels below current annual average air quality standards in the United States, Canada, and Europe at the time of completing the research. The current National Ambient Air Quality Standards (NAAQS) for PM_2.5_ is 12 μg/m^3^, although very recently, in March 2024, the U.S. Environmental Protection Agency lowered the annual average NAAQS to 9 μg/m^3^.^12^ The current 2020 Canadian Ambient Air Quality Standards for long-term exposure to PM_2.5_ is 8.8 μg/m^3^.^13^ The proposed new EU annual limit value is 10 μg/m^3^ for PM_2.5_, considerably more stringent than the current limit value of 25 μg/m^3^; this value is under final consideration by the European Parliament and the Council.^14^

A main goal of the HEI research initiative was to fund large studies to assess health effects of long-term exposure to low levels of ambient air pollution, including all-cause and cause-specific mortality and morbidity.^15^ Such studies were to analyze and evaluate ERFs for PM_2.5_ and other pollutants at levels currently prevalent in North America, Western Europe, and other high-income regions. A second goal was to develop statistical and other methodologies specifically suited to conducting such research, including the evaluation and correction of ERFs for exposure measurement error. The three study teams began the multiyear studies in 2016, and their work has now been reported in multiple peer-reviewed papers and HEI reports. All three studies met the main aims of the research initiative.

## 2 Methods

We provide an overview and synthesis of the results of this low-exposure epidemiology initiative, bringing in evidence from other key research that addresses whether associations with adverse health effects continue to be observed at current levels of air pollution, and that describes the shape of the ERF at those low levels. We discuss strengths and limitations of the research and remaining areas for future research. The evaluation of the three studies included here is based on an independent review by the HEI Low-Exposure Epidemiology Studies Review Panel (referred to as “Review Panel”). A key goal is to provide an overview of the remarkable body of work produced by the investigators, to make the findings readily accessible, and to emphasize future directions in research. We focus primarily on all-cause mortality as this outcome is most influential in terms of guiding regulation and associated cost-benefit analyses, and not subject to misclassification.

## 3 Summary of the Studies’ Approaches and Key Results

HEI published final reports of the studies in 2021 and 2022.^16−18^ First phase reports of the Canadian and U.S. work were published in 2019.^19,20^ We provide a summary of the studies’ approaches, key results, and their interpretation. We note if results are documented elsewhere than in the final HEI reports.

### 3.1 Canadian MAPLE Study

The Mortality-Air Pollution Associations in Low-Exposure Environments (MAPLE) study by Michael Brauer, University of British Columbia, and colleagues aimed to characterize the association between long-term exposure to outdoor PM_2.5_ and nonaccidental- and cause-specific mortality in a nationally representative sample of 7.1 million Canadian adults. The investigators assembled a cohort that combined three cycles (1991, 1996 and 2001) of the Canadian Census Health and Environment Cohort (Stacked CanCHEC). In addition, MAPLE included a Canadian Community Health Survey cohort composed of randomly selected participants (*N* = 540 900) who completed a health survey between 2001 and 2012 that captured additional individual information about lifestyle factors.

The investigators estimated PM_2.5_ exposures across North America from 1981 to 2016 at a spatial resolution of 1 km by 1 km using a method that incorporated satellite, ground monitor, and atmospheric modeling data. They estimated PM_2.5_ concentrations averaged over 10 years and linked estimates to the cohort using postal code of residence while accounting for address changes. O_3_ and O_*x*_ (O_3_ + NO_2_) estimates were derived from existing land use^21^ and chemical transport models^22,23^ and were used in copollutant models.

The investigators applied Cox proportional hazard models to estimate associations between PM_2.5_ exposure and mortality outcomes. They estimated the shapes of ERFs using restricted cubic splines with 3 to 18 knots, standard threshold models, and extended shape-constrained health impact functions (SCHIFs). The analyses were adjusted for the region of Canada, census year, and many individual- and area-level sociodemographic factors (Table 1).

**Table 1:** Key Study Characteristics of the Low-Exposure Epidemiology Initiative

study characteristics^a^	Canadian MAPLE study	U.S. Medicare study	European ELAPSE study	
			ELAPSE Pooled	ELAPSE Administrative
study population	Stacked CanCHEC (CanCHEC 1991, CanCHEC 1996, CanCHEC 2001), and CCHS	U.S. Medicare enrollees	15 smaller cohorts in 7 European countries	6 nation-wide cohorts and 1 city-wide cohort in Europe
sample size	7.1 million	68.5 million	325 000	28 million
number of deaths	1.3 million	27.1 million	47 000	3.6 million
study period	1991–2016	2000–2016	1985–2015	2000–2017
age	25+	65+	25+	30+
health outcomes	nonaccidental and cause-specific mortality	all-cause mortality	nonaccidental and cause-specific mortality, various morbidity outcomes	nonaccidental and cause-specific mortality
pollutants	PM_2.5_	PM_2.5_, NO_2_, and O_3_	PM_2.5_, PM_2.5_ composition, BC, NO_2_, and O_3_	PM_2.5_, PM_2.5_ composition, BC, NO_2_, and O_3_
exposure assessment	satellite-based model for North America	US-wide ensemble-based model using various machine-learning techniques	hybrid land-use regression model for Western Europe	hybrid land-use regression model for Western Europe
spatial resolution	1 km by 1 km	1 km by 1 km	100 m by 100 m	100 m by 100 m
spatial resolution exposure assignment to the cohort	residential postal code	residential zip code	residential address	residential address
residential mobility	yes	yes	not accounted for in the main analysis	not accounted for in the main analysis
temporal resolution	annual	daily	annual	annual
exposure window and lag time	ten-year moving average with a one-year lag	annual time-varying average, no time lag	annual average, no time lag	annual average, no time lag
covariate adjustment	stratified by sex, age, cohort, and recent immigrant status; adjusted for income adequacy quintile, visible minority status, Indigenous identity, educational attainment, labor-force status, marital status, occupation, and ecological covariates of community size, airshed (6 regions), urban form, and four dimensions of the Canadian Marginalization Index	stratified by sex, age, race-ethnicity, and Medicaid eligibility; adjusted for zip code-level information on SES, race, education, population density, and meteorological variables, and county-level smoking rate and BMI, region (4 areas), and calendar year	age (time axis), sex (strata), cohort (strata), calendar year of enrollment, smoking status, smoking intensity and duration current smokers, BMI, employment status, marital status, and area-level income	age (time axis), sex (strata), calendar year of enrollment, and cohort-specific individual and area-level SES information
co-pollutant analysis	two-pollutant models (O_3_ and O_*x*_ [O_3_ + NO_2_])	two- and three-pollutant models	two-pollutant models	two-pollutant models
study design	cohort-specific and pooled cohort analyses	open cohort analysis	pooled cohort analysis	cohort-specific analyses and meta-analyses
statistical methods	Cox proportional hazard models	Cox proportional hazard models, Poisson regression and three causal inference methods	Cox proportional hazard models	Cox proportional hazard models
PM_2.5_ mean exposure (μg/m^3^)	8	10	15	8 (Norway) to 19 (Belgium)
PM_2.5_ lowest exposure (μg/m^3^)	2.5 (min), 3.9 (P5)	2.8 (min)	3.2 (min), 8.6 (P5)	3.9 (Norway) to 15.6 (Belgium) (P5)

a MAPLE = Mortality-Air Pollution Associations in Low-Exposure Environments. CanCHEC = Canadian Census Health and Environment Cohort. CCHS = Canadian Community Health Survey. ELAPSE = Effects of Low-Level Air Pollution: A Study in Europe. Min = minimum. P5 = the fifth percentile.

**Figure 1:**
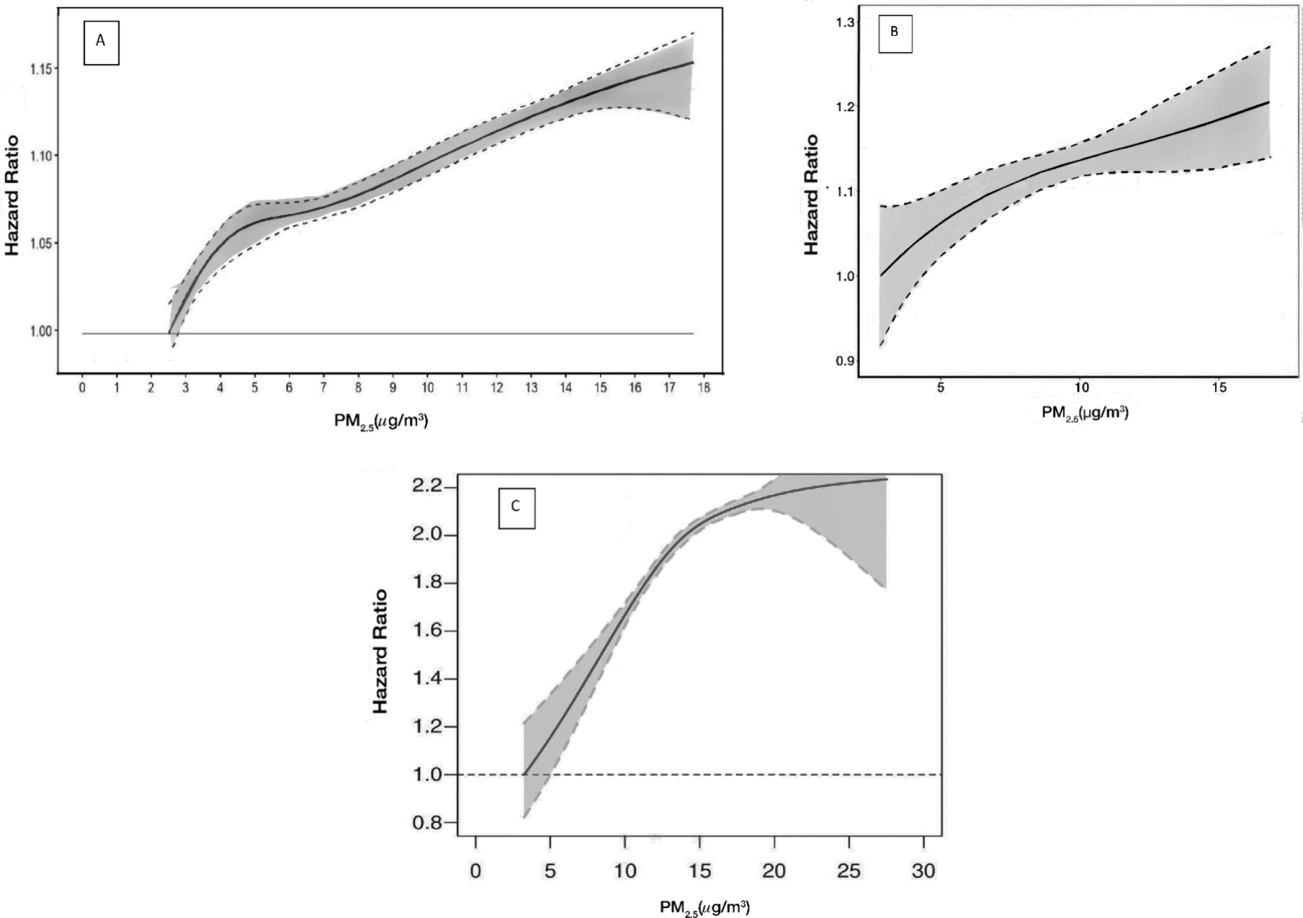
ERFs between long-term exposure to PM_2.5_ and all-cause or nonaccidental mortality in (A) the Canadian MAPLE study; (B) U.S. Medicare study; and (C) European ELAPSE Pooled cohort. Adapted with permission from HEI.

In the Stacked cohort, the mean estimate of PM_2.5_ exposure was 8 μg/m^3^. They reported good model performance, with an *R*^2^ of 0.81 when comparing PM_2.5_ concentrations estimated from the model with those measured at ground monitors across North America. The investigators reported an increased risk of nonaccidental mortality of 4% per 5-μg/m^3^ increase in PM_2.5_. They also reported that long-term exposures as low as 4 μg/m^3^ or even lower were associated with nonaccidental- and cause-specific mortality (Table 2). The ERF between PM_2.5_ and mortality was supra-linear, which indicates a larger relative effect per additional unit of exposure at low pollutant concentrations than at high concentrations (Figure 1). Results were similar compared to the full cohort when limiting the analysis to the subpopulation (87%) with PM_2.5_ exposure below the annual NAAQS of 12 μg/m^3^. In contrast, there was no association when limiting the analysis to the subpopulation (∼70%) exposed below 10 μg/m^3^. Furthermore, the effect estimate was smaller with adjustment for O_3_ or O_*x*_ (O_3_ + NO_2_), and notably different results were observed for the different regions of Canada, which could not be explained by differences in lifestyle factors, population characteristics, and healthcare access.

**Table 2:** Comparison of PM_2.5_ Findings for All-Cause or Nonaccidental Mortality with HEI-Funded Studies and Recent Systematic Reviews

					effect estimate^b^ (95% CI) PM_2.5_ per 5-μg/m^3^	
study^a^	cohort	statistical methods	study population	subpopulation	full population	subpopulation <12 μg/m^**3**^
Canadian MAPLE	Stacked CanCHEC	Cox	7.1 million	6.2 million	1.041 (1.036–1.047)	1.031 (1.024–1.038)
	1991 CanCHEC	Cox	2.5 million	NR	1.034 (1.026–1.042)	NR
	1996 CanCHEC	Cox	3 million	NR	1.037 (1.029–1.046)	NR
	2001 CanCHEC	Cox	3 million	NR	1.053 (1.042–1.064)	NR
	CCHS without lifestyle	Cox	540 900	NR	1.060 (1.028–1.093)	NR
	CCHS with lifestyle adjustments	Cox	540 900	NR	1.042 (1.010–1.075)	NR
U.S. Medicare		matching	68.5 million	38.4 million	1.033 (1.026–1.040)	1.127 (1.114–1.141)
		weighting	68.5 million	38.4 million	1.037 (1.032–1.043)	1.139 (1.120–1.159)
		adjustment	68.5 million	38.4 million	1.035 (1.030–1.040)	1.110 (1.085–1.137)
		Cox	68.5 million	38.4 million	1.032 (1.029–1.036)	1.169 (1.154–1.185)
		Poisson	68.5 million	38.4 million	1.031 (1.027–1.034)	1.158 (1.144–1.173)
ELAPSE Pooled		Cox	325 000	52 528	1.130 (1.106–1.155)	1.296 (1.140–1.474)
ELAPSE Administrative	Combined	Cox	28 million	4 million	1.053 (1.021–1.085)	1.095 (1.002–1.197)
	Belgian	Cox	5.5 million	14 395	1.023 (1.011–1.035)	0.970 (0.592–1.587)
	Danish	Cox	3.1 million	1.3 million	1.141 (1.118–1.164)	1.263 (1.212–1.315)
	Dutch	Cox	1.0 million	27 129	1.021 (0.999–1.044)	0.168 (0.039–0.733)
	English	Cox	1.4 million	266 377	1.023 (1.001–1.045)	1.080 (1.006–1.161)
	Norwegian	Cox	2.3 million	2.2 million	1.076 (1.066–1.086)	1.074 (1.064–1.085)
	Roman	Cox	1.3 million	49	1.066 (1.033–1.099)	NR
	Swiss	Cox	4.2 million	265 253	1.026 (1.015–1.038)	1.024 (0.983–1.067)
Chen and Hoek 2020	Systematic review	Primarily Cox	25 studies	9 studies	1.039 (1.032–1.047)	1.058 (1.037–1.080)^c^
Pope et al. 2020	Systematic review	primarily Cox	33 studies	NR	1.039 (1.027–1.051)	NR

a MAPLE = Mortality-Air Pollution Associations in Low-Exposure Environments. CanCHEC = Canadian Census Health and Environment Cohort. CCHS = Canadian Community Health Survey. ELAPSE = Effects of Low-Level Air Pollution: A Study in Europe.

b NR = not reported. Hazard ratios from fully adjusted models and converted to 5-μg/m^3^ to allow comparison.

c restricted to cohorts with a mean level <12 μg/m^3^

Long-term PM_2.5_ exposure was also associated with increased nonaccidental mortality in the individual CanCHEC cohorts and the Canadian Community Health Survey cohort (Table 2). In the survey cohort, adjustment for individual-level health behaviors attenuated associations, making them similar to associations in the Stacked cohort. The investigators hypothesized that after adjusting for the numerous individual- and community-level socio-demographic variables, lifestyle factors might not be important confounders at the low PM_2.5_ levels observed in this study population.

### 3.2 U.S. Medicare Study

The Medicare study by Francesca Dominici, Harvard T.H. Chan School of Public Health, and colleagues evaluated the risk of all-cause mortality associated with exposure to low concentrations of ambient air pollution in a cohort of 68.5 million older Americans enrolled in the U.S. Medicare program. The investigators used machine learning techniques to develop exposure models for daily PM_2.5_, O_3_ and NO_2_ covering the contiguous United States at a special resolution of 1 km by 1 km for the years 2000 to 2016. The exposure model inputs included monitoring data from the U.S. EPA Air Quality System, satellite observations, meteorological variables, land use data, and dispersion models. The investigators assigned the predicted annual average exposures to cohort participants’ residential zip code of residence for each year of follow-up (Table 1).

They developed and applied three causal inference methods that adjusted for confounding using generalized propensity scores by matching, weighting, and adjustment. They also applied two standard regression approaches, namely Cox proportional hazard models and Poisson models. All analyses adjusted for age, sex, race-ethnicity, Medicaid eligibility, and zip-code level information, including indicators of socioeconomic status (SES), county-level information on body mass index (BMI) and smoking, broad region, and calendar year. They utilized findings from a smaller Medicare cohort (Medicare Current Beneficiary Survey) that had individual lifestyle information available to assess the likely impact of having only a limited number of individual-level covariates in the main analysis; those results were reported in the Phase 1 report,^20^ with more details in Makar et al.^24^ and Di et al.^25^ Lastly, the investigators applied the newly developed generalized propensity scores matching method to estimate the shape of the ERF for each pollutant individually and with adjustment for the other two pollutants considered. The highest and lowest 1% of pollutant exposures were excluded to avoid instability at the boundaries of the exposure distribution.

The mean estimate of PM_2.5_ as assigned to cohort participants was 10 μg/m^3^. The investigators reported good model performance, with a cross-validation *R*^2^ of 0.86 for daily PM_2.5_ exposure predictions and less exposure error at low concentrations. The investigators reported increased risks of all-cause mortality of 3% to 4% per 5-μg/m^3^ increase in PM_2.5_ across the five approaches, with larger effect estimates for a subpopulation (∼56%) exposed below or equal to the annual NAAQS of 12 μg/m^3^ (Table 2). The U.S. Medicare study did not specifically investigate the subpopulation exposed below 10 μg/m^3^. The ERF was nearly linear at exposure below the PM_2.5_ NAAQS, with no evidence of a threshold (Figure 1). The investigators also reported associations between NO_2_ and O_3_ with mortality at higher concentrations. For NO_2_, associations below 53 ppb (approximately 100 μg/m^3^), the current annual NAAQS, were nonlinear and statistically uncertain. For O_3_, the ERF was almost flat below 45 ppb (approximately 88 μg/m^3^), showing no statistically significant association. Generally, adjusting for the other two pollutants slightly attenuated the effects of PM_2.5_ on all-cause mortality and slightly elevated the effects of NO_2_ exposure; results for O_3_ remained unchanged. Sensitivity analyses using the smaller Medicare cohort indicated that the inability to adjust for individual lifestyle factors (e.g., smoking) in the full cohort did not affect the results of the main analysis.

### 3.3 European ELAPSE Study

The Effects of Low-Level Air Pollution: A Study in Europe (ELAPSE) study by Bert Brunekreef, Utrecht University, and colleagues examined whether long-term exposure to low concentrations of ambient air pollution is associated with health effects in 22 European cohorts. Air pollution concentrations were estimated with a hybrid land-use regression model for Western Europe at a high spatial resolution (100 m by 100 m), combining monitoring data (e.g., from AirBase), land use data, satellite observations, and dispersion models for the year 2010. The investigators assigned the 2010 exposure estimates to the cohort participants using residential addresses at the year of recruitment. The investigators analyzed a pooled cohort that included 15 conventional epidemiological cohorts with detailed information available on lifestyle factors. Most of these cohorts had been analyzed previously as part of the European Study of Cohorts for Air Pollution Effects (ESCAPE) project.^26^ They also analyzed 7 very large administrative cohorts formed by linking census data, population registries, and death registries, but with less detailed covariate data (Table 1).

The investigators applied Cox proportional hazard models to describe associations between exposures to the pollutants and the health outcomes of interest. The team investigated the shapes of the ERFs using natural splines with two, three, and four degrees of freedom, with penalized splines, standard threshold models, and SCHIFs. They applied meta-smoothing approaches to obtain a meta-analytic ERF for the administrative cohorts and reported those in Stafoggia et al.^27^ All analyses were adjusted for age, sex, calendar year of enrollment, and selected individual and area-level SES information. The pooled cohort was also adjusted for lifestyle factors; the administrative cohorts explored indirect adjustment approaches to adjust the risk estimates for these missing covariates. Finally, the pooled cohort analysis was stratified by subcohort to account for differences in baseline hazards between the cohorts. They also applied causal inference methods that adjusted for confounding using generalized propensity scores by weighting in a subset of the data, with additional HEI-funding, and published separately from the main report.^28^

Mean exposure estimates for PM_2.5_ across the various cohorts ranged from 8 μg/m^3^ (Norway) to 19 μg/m^3^ (Belgium); the mean exposure in the pooled cohort was 15 μg/m^3^. The final, Europewide hybrid LUR exposure models explained 66% of the variability in concentrations of PM_2.5_, with good spatial and temporal stability. For both approaches (pooled and administrative cohorts), the investigators reported that exposure to PM_2.5_, BC, and NO_2_ was associated with nonaccidental, cardiovascular, respiratory, and lung cancer mortality. They also reported inverse associations between O_3_ and all causes of death examined in single-pollutant models, related to the negative correlation between O_3_ and the other pollutants. The investigators reported an increased risk of nonaccidental mortality of 13% and 5% per 5-μg/m^3^ increase in PM_2.5_ in the ELAPSE pooled and the ELAPSE administrative studies, respectively (Table 2). The estimated risks associated with exposure were generally greater in the pooled cohort than in the administrative cohorts. High heterogeneity of the effect estimates was reported for mortality across the administrative cohorts. Indirect adjustment for smoking and BMI had a negligible effect on the main results for nonaccidental mortality.

In two-pollutant models, the risk estimates for mortality were attenuated but remained elevated for PM_2.5_ and NO_2_ in the pooled cohort; in the administrative cohorts, the risk estimate for PM_2.5_ was attenuated to unity when adjusted for NO_2_, whereas the mortality association for NO_2_ and BC remained stable after adjustment for PM_2.5_. In two-pollutant models for O_3_, associations were attenuated but remained negative in the pooled cohort, whereas O_3_ attenuated to unity in the administrative cohorts. Note that two-pollutant models of BC and NO_2_ were not interpreted because of high correlation between the two.

For both approaches, the shape of the ERFs with nonaccidental mortality showed steeper slopes at lower exposures for PM_2.5_ (Figure 1), BC, and NO_2_, with no evidence of a threshold. However, the shape of the ERF differed substantially among the administrative cohorts. The associations for PM_2.5_ with nonaccidental mortality were stronger when the analyses were restricted to the subpopulations (16% and 14%, respectively, for the pooled and the administrative cohorts) below the annual NAAQS of 12 μg/m^3^. Stronger associations were also observed in the subset (6% and 7%, respectively) below 10 μg/m^3^. Finally, they found similar results using causal inference methods, both in the pooled cohort and in the single administrative cohort that was analyzed with these methods.^28^

The investigators conducted some additional work related to PM composition and sources after the publication of the final report, with additional HEI funding. In short, all eight PM components — copper (Cu), iron (Fe), potassium (K), nickel (Ni), sulfur (S), silicon (Si), vanadium(V), and zinc (Zn) — were associated with nonaccidental mortality in single-pollutant models, but estimates for most of the components were attenuated to unity after adjustment for PM_2.5_ or NO_2_.^29,30^ Generally, linear or supra-linear ERFs were reported for the components and mortality in the ELAPSE pooled cohort.^29^ Furthermore, in subsequent source apportionment analysis in the ELAPSE pooled cohort, they identified five sources: traffic, residual oil combustion, soil, biomass and agriculture, and industry.^31^ In single-source analyses, all identified sources were significantly positively associated with increased nonaccidental mortality risks. In multisource analyses, associations with all sources were attenuated but remained statistically significant with traffic, residual oil combustion, and biomass and agriculture. The largest effect estimate per interquartile increase across the five identified sources was observed for the traffic component. On a per unit mass basis, the effect estimate for residual oil-related PM_2.5_ was the largest and substantially greater than that for generic PM_2.5_ mass.^31^ The PM component analyses were hampered by the moderate performance of the models for PM composition, the high correlations of concentrations among some of the components and, in some instances, small within-cohort exposure contrast.

### 3.4 Harmonized Analysis Across the Canadian, U.S., and European Studies

To increase comparability across studies, a harmonized analysis for PM_2.5_ and mortality was conducted across studies using the same exposure model, outcome definition, age of the population, covariates, and statistical models as much as possible.^32^ Participants aged 65 years or older in six administrative cohorts in Europe, the U.S. Medicare study, and the Stacked CanCHEC cohort were included in this analysis, resulting in a very large study population of 81 million participants. For this harmonized analysis, the investigators used annual PM_2.5_ estimated from the satellite-based exposure model from the Canadian MAPLE study, which is a model with global coverage, including Europe.^33^ Annual PM_2.5_ exposures were estimated for study participants at the residence based on zip or postal codes (Medicare and MAPLE) or addresses (ELAPSE). In ELAPSE and Medicare, annual average PM_2.5_ concentrations were assigned to individuals for that calendar year, whereas in MAPLE a ten-year moving average of PM_2.5_ with a one-year lag was assigned. All-cause mortality was used as the health outcome (including accidental/trauma mortality) because the Medicare study included information only on all-cause mortality.^32^

The investigators applied standard Cox proportional hazard models to describe associations between PM_2.5_ exposure and all-cause mortality in the European and Canadian studies; Poisson regression was used in the U.S. study instead for computational efficiency given the large size of the Medicare cohort. The teams investigated the shapes of the associations using extended SCHIFs, the same method used in the Canadian MAPLE study. The analysis was adjusted for individual-level age, sex, cohort (for CanCHEC), follow-up year, individual-level SES or ethnicity, area-level SES covariates, and broad regional indicators to account for residual spatial variation.^32^

Positive associations were reported in all three studies in the harmonized analysis but were slightly smaller than those in the main analysis (Table 2). In the harmonized analysis, hazard ratios and 95% confidence intervals associated with a 5-μg/m^3^ increase in PM_2.5_ exposure were 1.042 (1.015, 1.069) for the six ELAPSE administrative cohorts, 1.039 (1.032, 1.046) for the stacked CanCHEC, and 1.025 (1.021, 1.029) for Medicare. The shape of the ERF differed across the eight cohorts, but generally showed associations down to the lowest observed PM_2.5_ levels (4 μg/m^3^). The combined ERF showed an increased risk albeit with wide uncertainty at lower concentrations (<7 μg/m^3^) due to the variation in the ERF in the U.S Medicare (sublinear), and the CanCHEC and Norway studies (both supra-linear). The Medicare study had the largest weight (44%) in the combined ERF.^32^ Note that the sample size in the U.S. Medicare study differed in the harmonized analysis (74.5 million) compared to the Phase 2 report^18^ (68.5 million) because the covariate adjustment sets are different for these two analyses. In the Phase 2 report,^18^ they adjusted additionally for smoking rate, BMI, and meteorological variables. The variability in the magnitude and shape of the association across the Canadian, U.S. and European studies was reduced only slightly in the harmonized analysis.

### 3.5 Comparison with Other Research

The results generally corroborate those of prior studies showing increased risk of death for all-cause, respiratory, and cardiovascular-related mortality below current PM_2.5_ standards (Table 2). They add to the growing number of studies that suggest the shape of the ERF is either linear or supra-linear at lower PM_2.5_ concentrations, with no evidence of a threshold.

For example, the systematic review underpinning the 2021 WHO Air Quality Guidelines^5^ for long-term exposure to PM_2.5_ and all-cause mortality reported a summary estimate of 1.04 per 5-μg/m^3^ with a confidence interval of 1.03, 1.05, based on 25 studies.^9^ The summary estimate tended to be larger in the nine studies with a mean PM_2.5_ concentration below 12 μg/m^3^. The lowest value reported as a fifth percentile of population exposure from studies included in the Chen and Hoek^9^ meta-analysis was 3 μg/m^3^. Furthermore, most studies that analyzed the ERFs found no evidence of a threshold and showed linear or supra-linear functions.^25,34−39^ Another review reported a summary estimate of 1.04 per 5-μg/m^3^ with a confidence interval of 1.03, 1.05, based on 33 studies.^11^

In a meta-regression of data from 53 cohort studies, the shape of the ERF was investigated by applying unrestricted smoothing splines.^40^ Those authors reported evidence for an effect on mortality that extended to PM_2.5_ levels below 10 μg/m^3^ and observed a supra-linear association for nonaccidental mortality.^40^ Furthermore, in an analysis using SCHIFs and data from 41 cohorts, a supra-linear association was observed as well between PM_2.5_ and mortality.^41^ Note that many of these analyses draw on data from the same cohorts.

Also, the findings for the other pollutants included in the three studies broadly agree with prior research,^10^ except for the unexpected finding in the European ELAPSE study of inverse associations between O_3_ and the risk of mortality and morbidity. In ELAPSE, O_3_ was highly (negatively) correlated with PM_2.5_ and NO_2_ in contrast to the U.S. Medicare study for which positive correlations and associations were reported. These inverse associations in ELAPSE, however, were also found in the two-pollutant models in the pooled cohort but not the administrative cohorts. Also, the O_3_ associations remained inverse when O_3_ exposure was aggregated at a larger spatial scale (50 by 50 km), which would be more comparable to the exposure assignment in the North American studies. The exposure range for O_3_ was somewhat lower in ELAPSE than in the North American studies, potentially limiting the European analysis. Findings from subsequent analyses carried out by the ELAPSE investigators showed that the inverse association with O_3_ in the pooled cohort was attenuated when the large Austrian cohort (VHM&PP) that experienced the highest O_3_ concentrations was not included, but only when coupled with adjustment for any of the copollutants. With additional adjustment for noise, the inverse association was attenuated to unity.^42^

For NO_2_, associations below 53 ppb (approximately 100 μg/m^3^) were nonlinear and statistically uncertain in the U.S. Medicare study, whereas the ELAPSE study reported associations with steeper slopes at lower exposures, with no evidence of a threshold. Moreover, the NO_2_ findings in ELAPSE remained stable in the two-pollutant models, suggesting that the positive association may reflect, at least in part, an independent effect of NO_2_ itself. NO_2_ originates largely from motor vehicles in cities, and the ELAPSE study captured local gradients at a finer scale than the U.S study, as discussed below. The finer scale estimates reduce measurement error, which is important for pollutants such as NO_2_ that vary substantially in space. While evidence on the effect of NO_2_ has strengthened in recent years,^2,10,43,44^ a key question that remains largely unresolved so far is whether NO_2_ has independent effects or whether it is merely an indicator of traffic-related air pollution.

## 4 Discussion

The simultaneous funding and the collaborations among the investigators created by HEI fostered synergies among the teams, facilitating methodological developments and harmonization for pooled analyses. The incorporation of cohorts with individual covariate information and very large administrative cohorts (though with less detailed information) provided new insights as to the merits of both approaches. Particularly strong aspects of the studies included the unprecedentedly large populations (7–69 million) with national representativeness, and with less risk of selection bias and loss to follow-up. Additional strengths were the state-of-the-art exposure assessment methods with greater spatial resolution than used previously, and thorough statistical analyses with novel methods to assess the associations between air pollution exposure and mortality. The Review Panel appreciated that some of the exposure and cohort data have been made publicly available, thus facilitating transparency and reproducibility. Dozens of peer-reviewed papers have been published by the study teams, many in high-impact journals such as *The New England Journal of Medicine* and *The British Medical Journal*.^25,42^ All three studies addressed critical research gaps in understanding the health effects of low-level ambient air pollution and provided policy-relevant science.

Despite these many strengths, the Review Panel noted some limitations of the approaches used, such as the validity of the exposure estimates in rural areas, zip code-level aggregation in the U.S. analysis, and the potential influence of PM_2.5_ components. These and other aspects of the study designs and approaches and the interpretations of the findings are discussed in the following sections.

### 4.1 State-of-the-Art Exposure Assessment Methods

The development of state-of-the-art exposure assessment methods was an impressive achievement of each of the three studies because of (1) the large geographic scope covered by the exposure models (e.g., Canada is ∼10 million km^2^; the entire continental U.S. is ∼8 million km^2^); (2) the enormous amount of data and the variety of data sets assembled; and (3) the immense computational requirements. These exposure models allowed the investigators to assign exposure estimates to cohort participants in all locations, including those in rural and remote areas where there are few or no pollution monitors. Although all the exposure models were extensively validated and found to perform well, the Review Panel had concerns about the quality and accuracy of the estimates for rural areas because there are few or no pollution monitors in those areas. Generally, existing monitors are located for compliance with standards, and are therefore placed in more populated, urban areas where air pollution concentrations are higher. Consequently, rural areas—where population densities and pollutant concentrations are lower—are not monitored as intensively. Thus, the models can be more prone to larger errors in estimates for rural areas, and those estimates cannot be validated as well as at other locations. Given that relatively few people live in these areas, the exposure errors might not have much influence on the overall exposure estimates or the main epidemiological analyses. If these rural populations represent a sufficiently large portion of those with the lowest exposures, however, then the errors introduced here could be particularly influential at the low end of the ERF and on subsequent epidemiological analysis.

Generally, the Review Panel was impressed with the generation of models at the relatively fine spatial scale of 1 km by 1 km (U.S. and Canada) and even finer, at 100 m by 100 m (Europe). The 1 km by 1 km spatial resolution might be sufficient for PM_2.5_ because of the largely regional spatial distribution of PM_2.5_ with limited local variability. Those models, however, do not capture local gradients in concentrations, such as those along roadways or near major point sources, which can be substantial for certain pollutants such as NO_2_, BC, and O_3_. Those local gradients are better captured in the European ELAPSE model, though admittedly, not fully even with a 100 m by 100 m resolution.

In the European ELAPSE study, predicted exposures were assigned to cohort participants’ residential addresses. Such information is very difficult to obtain in North America. Therefore, as noted above, the Canadian and U.S. teams had to aggregate the pollution estimates to the geographic scale of postal codes (Canada) or zip codes (U.S.) for estimating participants’ long-term exposures. In Canadian urban areas, a residential postal code centroid is typically within ∼500 m of a person’s home, whereas in rural areas the location for a given postal code is typically accurate within about 1–5 km.^45^ Reassuringly, in the Canadian MAPLE study, associations were not sensitive to PM_2.5_ exposure assignment at different spatial scales (1, 5, and 10 km), as described in sensitivity analyses presented in the Phase 1 report.^19^ U.S. zip codes vary substantially in size based on population density. Zip codes are on average 24 km^2^ in Los Angeles County, California, and 268 km^2^ in the state of Texas. As such, the assignment of PM_2.5_ exposure to the relatively coarse zip code level in the Medicare study might result in more measurement errors compared to the assignment of postal code level in MAPLE and residential address level in ELAPSE. This issue might imply even greater exposure error in rural areas, which typically also have the lowest concentrations.

It is often assumed that the exposure measurement error would likely bias the estimated PM_2.5_-mortality association toward the null.^46^ Indeed, in the few epidemiological studies that corrected for exposure measurement error, correction resulted in small increases in the magnitude of the association, and its standard error.^47^ However, because the measurement error may be complex and not purely classical or Berkson in structure, the nature of the potential bias cannot be fully known.^48^ No study has explicitly focused on measurement error arising in low concentrations of air pollution specifically or the potentially differing measurement error across the distribution of PM_2.5_ concentrations.^49^ The evaluation and correction of health estimates for exposure measurement error was not comprehensively assessed, despite being specifically listed in the method development aim of the HEI research initiative. For the U.S. Medicare study, Dominici et al. conducted some work in this area, as reported in the Phase 1 report.^20^ The investigators developed a regression calibration approach under a causal inference framework for categorical exposures and applied the approach using Medicare data in the Northeastern United States. When accounting for exposure error, they found there was a larger and still statistically significant association between exposure to PM_2.5_ and mortality, although with larger confidence intervals.^20,50^ For the ELAPSE study, Brunekreef et al. explored a regression calibration approach in the pooled cohort that accounted only for classical errors in the exposure model.^17^ Application of regression calibration resulted in very small changes in the effect estimates and the confidence intervals.^17^ An overarching challenge to measurement error correction is the need for a gold standard, which is long-term personal exposure from outdoor sources. Such a gold standard is nearly impossible to obtain. How to propagate exposure measurement error into health effects estimation in long-term air pollution and health studies remains an area of active research.

### 4.2 Rigorous Statistical Analyses with Novel Methods

The Review Panel was impressed with the rigorous analyses in all three studies, including the numerous sensitivity and subset analyses conducted and generally found them helpful in supporting the robustness and interpretation of the findings. Broadly, these analyses related to restricting participants with mean exposures below selected concentrations, applying different approaches to exposure specification (e.g., estimating exposures only at baseline versus using time-varying estimates, or exploring different time windows), exploring sensitivity to confounder control (e.g., adjusting for additional confounders), estimating and accounting for effects of copollutants, and examining various approaches to characterize ERFs (e.g., splines, SCHIFs, or threshold models).

The use of SCHIFs was considered a valuable addition. They place constraints on the shape of the ERF to be consistent with known biological ERFs, for example, not allowing multiple wiggly curves. Those constraints make SCHIFs potentially more suitable for use in burden and health impact assessments. SCHIFs were developed by Nasari and colleagues^51^ and then generalized as the Global Exposure Mortality Model by Burnett and colleagues.^41^ The Review Panel was unclear about the estimated uncertainty at the low end of the curve for the (extended) SCHIFs applied in the European and Canadian studies, and further refinement seems to be warranted. Despite this, the standard statistical approaches to characterize ERFs that were used reached similar conclusions, which was reassuring.

In all three studies, standard Cox proportional hazard models that adjusted for individual-level and area-level confounders were applied. Moreover, extensive sensitivity analyses were performed to check for potential residual confounding from omitted covariates, such as lifestyle information. The great majority of cohort studies on air pollution and mortality to date have applied Cox proportional hazard models.^9,10^ More recently the use of causal inference methods has gained popularity in environmental health and air pollution epidemiology.^52−54^ For the U.S. Medicare study, the investigators developed and applied three causal inference methods using generalized propensity scores. Also, in the European study a causal inference approach in a subset of the data was used. In both studies, the causal inference findings were compared to the results from standard Cox and Poisson models, because ultimately all approaches are attempting to get an unbiased estimate for a presumed causal relationship. Each approach individually has relative strengths and limitations, but together they allowed the investigators to carry out a thorough and robust investigation.

An attractive feature of causal inference methods is that they attempt to mimic randomized clinical trials in which participants are randomly assigned to an exposed group and a reference group, such that potential confounders that are known to affect participants’ mortality can be balanced between the two groups. Because propensity score methods are typically applied to a categorical exposure (i.e., an exposed versus a less exposed or unexposed reference population), the U.S. investigators developed and implemented novel generalized propensity score approaches to accommodate the continuous air pollution exposures in the study.^55^ This same approach was implemented in the European study.

Although the development and application of causal inference methods was a major achievement in the U.S. Medicare study, the Review Panel cautioned against unrealistic expectations. Further development of causal inference methods in air pollution research is clearly needed, such as accounting for exposure measurement error for continuous exposures and capturing “spillover” effects—although recent promising advances have been made.^56,57^ The causal modeling approaches in the U.S. Medicare study are also limited by the underlying data that uses spatially aggregated estimates of exposure and of several potential confounders (e.g., smoking). Only limited information was available at the individual level, and smoking information was available at the county level. As a counter to these concerns, the investigators found that results were not sensitive to the omission of several individual-level confounders using a nationally representative subsample of Medicare participants with individual information on risk factors. Indeed, those findings in the Phase 1 report^20^ generally support the validity of the approach to covariate adjustment taken in the final analyses presented here.

### 4.3 Difficulty Interpreting the Subgroup Analyses at Concentrations below Standards

Larger effect estimates were reported for the U.S. and European subpopulations at concentrations exposed below or equal to the annual PM_2.5_ NAAQS of 12 μg/m^3^, consistent with the near linear (U.S.) or supra-linear (Europe) ERF reported in the full population. Although subgroup analysis is helpful, restricting the analysis to a subset of the data has some interpretational limitations because the subpopulation exposed to low levels of PM_2.5_ may not have the same characteristics as the full study population. It is important to acknowledge that the European subgroup analyses were based on smaller numbers of cohorts that were less heterogeneous. The analysis at the lowest concentrations of PM_2.5_ (below 10 μg/m^3^) included data primarily from Norway and Stockholm, and sample size was limited. Hence, there remains limited evidence for associations at the lowest PM_2.5_ concentrations in ELAPSE. For the Medicare cohort a near linear ERF was observed for the full population while the effect of PM_2.5_ was greater in the lower exposure subgroup. This finding could reflect greater susceptibility of this subgroup in comparison with the full cohort. The subpopulation exposed to 12 μg/m^3^ or below excluded participants in large areas of the Eastern United States and likely excluded most people in most major cities. Whereas the main analysis describes the risk for the elderly U.S. population as a whole, the subgroup analysis to some extent reflects the risk for those in smaller towns and rural areas. This population tends to be of lower SES, with poorer health behaviors, limited access to health services, and higher prevalence of diabetes or other comorbidities, which might also increase susceptibility to the effects of exposure.^58,59^

It was somewhat puzzling that associations were similar in the Canadian study when limiting the analysis to the large subgroup (∼87%) with PM_2.5_ exposure below 12 μg/m^3^ compared to the full cohort, whereas a supra-linear ERF was reported in the full population. Hence, stronger effect estimates were expected in this subgroup. Furthermore, there was no association when limiting the analysis to the very large subpopulation exposed below 10 μg/m^3^, an inconsistency that is not readily explained.

### 4.4 Differences in Associations Across Populations or Locations

Although all three studies documented positive and significant associations between mortality and PM_2.5_ concentrations below current standards, substantial heterogeneity was found within and across studies both in the magnitude and shape of the association (Table 2 and Figure 1).

Different results were observed for the different regions of Canada, with positive associations in four broad regions (East Central, Southern Atlantic, Western, and Northern regions, 81% of the full population), and inverse associations in the others (Prairie and West Central regions). Note that the positive effect estimates between PM_2.5_ and nonaccidental mortality were relatively small (HRs ∼ 1.03 per 5 μg/m^3^) but significant for East Central (59% of the full population) and Western (12%) regions. In contrast, the positive effect estimates between PM_2.5_ and nonaccidental mortality were very high (HRs ∼ 1.18) for the two regions, Southern Atlantic and Northern regions (10%), with the lowest meanPM_2.5_ estimates. Inverse associations ranged from HRs ∼ 0.90 to 0.95. Those differing results were not explained by lifestyle factors, population characteristics or healthcare access. The variation in results may reflect underlying differences in air pollutant mixtures not characterized by PM_2.5_ mass concentrations or the included gaseous pollutants, namely O_3_ or O_*x*_. Beyond region, no other important effect modifiers were identified in the MAPLE study.

Moreover, the heterogeneity in the shapes of the ERFs in the various ELAPSE cohorts was not explained well beyond acknowledging that the cohorts differed in mean exposures. Some heterogeneity of the findings is expected, however, given the diversity of the cohorts, particularly in Europe. Note that in all ELAPSE administrative cohorts, the age of the population at baseline (<65 versus ≥65 years) was identified as an effect modifier, with stronger associations for the population <65 years and less heterogeneity of effect estimates in that group compared to the full population. No effect modification was observed in the ELAPSE pooled cohort. In the U.S. Medicare study, effect modification was reported for PM_2.5_ and mortality, specifically larger effect estimates for male, Black, Asian, and Hispanic subgroups in the Phase 1 report.^20^ Moreover, in a recent analysis using Medicare data, the investigators reported steeper ERFs for PM_2.5_ and mortality for Black persons than for white persons (regardless of income) albeit with overlapping confidence intervals, and for Black higher-income persons than for white higher-income persons.^60^

Heterogeneity is likely due to a combination of differences in methodology, concentration ranges and composition of PM_2.5_ or other copollutants, population characteristics, geographical location, and time periods. The meta-regression by Vodonos and colleagues^40^ suggests that, in particular, the degree of confounder adjustment, the average pollution level and the age of the population contributed to the heterogeneity in effect estimates of PM_2.5_ across studies worldwide.

In the recent systematic reviews of long-term exposure to PM_2.5_ and NO_2_ and the effects on mortality, a high degree of heterogeneity of the findings was also observed; a finding expected given the wide diversity of studies included from across the globe.^9,10^ Notably, heterogeneity especially remained within the large group of North American studies, and meta-regression exploring location, sex, age, and average pollution level did not explain the sources of high heterogeneity between studies. However, little effect-modifier information was available limiting the meta-regression.^9^ Similar to the findings of HEI’s low-exposure epidemiology initiative, most of the heterogeneity was due to variation in the magnitudes of the positive association across studies, not in the direction of the association (negative or positive).^9^

Somewhat surprisingly, heterogeneity was only reduced slightly in the harmonized analysis. Admittedly, some study characteristics were not harmonized—such as the spatial resolution of the exposure assignment to the cohorts, and the exposure windows and lag time—which may contribute to the remaining heterogeneity in the observed effect estimates and the different shapes of the ERFs. In addition, in the harmonized analysis the investigators were not able to adjust for individual lifestyle factors such as smoking because of the lack of information in the cohorts. However, all three studies provided evidence that lifestyle factors such as smoking might not be important confounders or effect modifiers in the study populations. There is often an implicit assumption that lack of adjustment for individual level confounders such as smoking would lead to an overestimation of air pollution risks, although this assumption has been refuted previously.^40^ Also, in the Canadian MAPLE and European ELAPSE cohorts, either similar (MAPLE) or smaller effect estimates (ELAPSE) were reported in the administrative cohorts compared to the smaller survey cohort and ELAPSE pooled cohort that had individual lifestyle information available. In the U.S. Medicare study, smoking was found to be correlated only weakly with air pollution exposure conditional on the other covariates included in the model.^25^ In recent systematic reviews of the association between PM_2.5_ and mortality, the meta-analytical effect estimates were not affected by excluding administrative cohorts that did not have individual lifestyle data available,^9,11^ implying that lack of data on smoking may not be critical.

## 5 Providing Science Relevant for Regulation and Burden Assessment

All three studies addressed critical research gaps in our understanding of the health effects of low-level ambient air pollution. Regulators want to know whether tightening PM_2.5_ standards below current levels might benefit public health and to what extent. Canada was an especially ideal setting to address these research gaps because the country typically has some of the cleanest ambient air quality globally. Indeed, half of the population in the Stacked CanCHEC cohort was exposed to mean PM_2.5_ levels below 8 μg/m^3^. The average PM_2.5_ exposures in the United States (10 μg/m^3^) and the European study (8–19 μg/m^3^, depending on the cohort) were somewhat higher. Even those levels were nevertheless lower than those seen in most prior studies,^9^ enabling the three study teams to evaluate the shape of the ERF between air pollution and health effects at the low end of the global exposure range.

The study findings inform regulatory decision-making in North America, Europe and around the globe. The Medicare and MAPLE studies were featured prominently in the Supplemental PM Integrated Science Assessment^4^ and Policy Assessment^61^ upon which the new U.S. NAAQS decisions are based. The U.S. Medicare study played a key role in informing the new PM_2.5_ NAAQS of 9 μg/m^3^ in part because it was the largest and most comprehensive study to date.^12^ The Medicare study^18,62^ was used in the policy assessment to calculate the expected mortality reductions in the U.S. population aged 65 or older for various alternatives to the annual PM_2.5_ NAAQS.^61^ Moreover, the Phase 1 study^20,25^ was also used to evaluate the environmental justice implications of the new PM_2.5_ NAAQS.^61^

The European Commission relies on WHO for science assessments. The European Commission’s proposal to revise the Ambient Air Quality Directive was heavily informed by the 2021 WHO AQG, including their accompanying impact assessment.^14,63^ The systematic reviews underpinning the 2021 WHO AQG were published in 2020 and included health studies from across the globe available until September 2018.^9,10^ Early results from the U.S. and Canadian study teams were included in those reviews.^25,38^ Furthermore, the European Commission conducted additional analyses using ELAPSE to estimate the influence of the choice of the ERF on mortality in the impact assessment.^64^ The use of ELAPSE resulted in higher attributable mortality estimates,^63^ indicating that the current health burden of PM_2.5_ air pollution may be underestimated in Europe.

Further evidence that the current health burden of PM_2.5_ air pollution may be underestimated was provided by a recent analysis from the Canadian team. They integrated the findings from MAPLE to refine the shape of a previously published global ERF for outdoor PM_2.5_ and mortality^65^ at the low end of the exposure distribution, as far down as 2.5 μg/m^3^.^66^ Use of the revised ERF increased the number of attributable deaths by 1.5 million each year globally compared to previous estimates, with larger underestimation of attributable mortality occurring in countries with lower PM_2.5_ concentrations and higher incomes.^66^ While many uncertainties remain, and more epidemiological studies are needed in very clean environments to corroborate the results, Weichenthal et al.^66^ clearly documented that the shape of the ERF between PM_2.5_ and mortality at low levels has a marked impact on global estimates of annual mortality attributable to PM_2.5_.

## 6 Remaining Areas for Further Research

HEI seeks to embark on the next stage of innovative and policy-relevant science on PM and its health effects, integrating valuable lessons learned from this research initiative into the new research. We describe below several areas of potential interest; some were discussed in a workshop that HEI held to inform the next stage of research in December 2023.

### 6.1 What is the Influence of PM Components?

As PM_2.5_ is a complex mixture that varies across both space and time, it is perhaps not surprising to observe differences in the magnitude and shape of the association simply because populations are not exposed to identical particles (despite similarity in PM_2.5_ mass concentrations and adjustments for NO_2_ and O_3_). Many features related to chemical composition, size, and other physical and biological properties of particles could be relevant.^67^ Sources and composition of PM_2.5_ mass vary across regions.^68,69^

Because the composition of PM is complex, there has long been a question as to whether some components of the PM mixture are of greater public health concern than others. Obtaining evidence indicating that specific PM characteristics drive risk would help focus efforts to reduce human exposure by enabling the control of those sources that contribute most of the toxic components in the PM mixture. In July 1997, the U.S. EPA, under the auspices of the federal Clean Air Act, established—for the first time—the NAAQS for PM_2.5_. A committee of the National Research Council was charged in 1998 with providing guidance to an extensive U.S. EPA research portfolio “to reduce uncertainties in the scientific evidence” underpinning the standards. One of the key research priorities identified was the question related to PM components and their relative toxicity, but only modest progress was made on those questions over the years per the conclusion of the committee in its final report—despite much, albeit fragmented, research.^70^

Subsequently, HEI supported the National Particle Component Toxicity (NPACT) Initiative, which involved coordinated epidemiological and toxicological studies to evaluate the relative toxicity of various chemical and physical properties of PM.^71,72^ The results indicated that component composition influences risk for health effects, but that there was no “silver bullet” at the time to guide regulatory efforts pointing to specific components or sources of PM_2.5_ as being more or less toxic. Additionally, the HEI NPACT Review Panel concluded that “the current practice of setting air quality standards for PM mass as a whole likely remains an effective approach to protecting public health”.^73^ Recently, the WHO concluded that insufficient data are available to provide recommendations for AQG for specific types of PM, notably black/elemental carbon, ultrafine particles, and sand and dust storms.^5^ WHO did, however, provide “good practice statements” for those other PM types geared toward additional monitoring, mitigation, and epidemiological research.^5^

The question related to relative toxicity of PM components has not gone away, and in fact is becoming even more important because of the increasing implementation costs for meeting stringent standards. The lack of routine ambient monitoring data on particle characteristics in many regions across the world hampers such research. Even in high-income countries where PM composition is monitored—as in the United States through its Chemical Speciation Network since 2000—the monitoring networks have limited spatial coverage, typically with few stations in suburban and rural locations, and insufficient density to capture small-scale variation of PM components. The research is also hampered by the high correlations among some particle components, and potential nonlinear interactions among components in relation to health outcomes. Exposure measurement and exposure modeling errors are additional complications.^74^ The development of multipollutant statistical approaches remains an active area of research, and many advanced approaches have been developed, particularly for omics analyses and in studies of the exposome.^75−77^ If greater success is to be achieved in characterizing the effects of different PM components and sources, advanced approaches and additional measurements will be needed so that exposure at the individual or population level can be assessed more accurately. An enhanced understanding of exposure and health will be needed before there is general agreement that regulations targeting specific sources or components of PM_2.5_ will protect public health more effectively than continuing to follow the current practice of targeting PM_2.5_ mass as a whole.^71^

Ultimately, more work is needed to understand the specific components and properties of PM_2.5_ that determine health effects before we can arrive at a more complete understanding of the shape of the ERF at low concentrations. Given the heterogeneous nature of PM_2.5_, there is no reason to believe that a single shape is appropriate for all locations and populations as spatial differences in components and sources likely play an important role in determining the shape of these associations.

### 6.2 PM in a Rapidly Changing Climate and Transportation Landscape

Another reason why the question of relative toxicity remains important relates to a rapidly changing climate and a changing transportation landscape resulting in PM from nontailpipe emissions and wildfires.^78,79^

Interest in the contribution of nontailpipe emissions to air quality and health is increasing across the globe given the push toward electrification of the vehicle fleet and that regulations continue to be targeted almost exclusively on tailpipe emissions.^80^ Nontailpipe emissions comprise particles in a broad range of sizes—including the coarse, fine, and ultrafine ranges—but compared with tailpipe PM emissions, they are generally in the larger size range and have less carbonaceous material and a higher metallic content.^81,82^ Hybrid and electric cars might produce greater amounts of tire wear because they are heavier and have more torque than internal combustion engine cars, although the use of regenerative braking would likely reduce both brake and tire wear by reducing slippage between surfaces (e.g., at the tire-road interface). However, the estimates of such emissions and experimental data vary widely.^83−85^ More research is needed to evaluate real-world exposure indicators of nontailpipe PM emissions from motor vehicles and to assess the effects of such emissions on air quality, exposure, and health. This research need was also flagged in HEI’s systematic review on the health effects of long-term exposure to traffic-related air pollution, which identified very few long-term health studies on nontailpipe PM indicators.^44,86^

Wildfire smoke is an increasingly important source of ambient PM_2.5_ in regions where emissions from major air pollution sources including transportation and power generation are declining. Wildfires are increasing in size and frequency worldwide, due in part to the hotter and drier conditions caused by human-induced climate change.^87^ Projections indicate that the risk of wildfires will continue to increase in most areas of the world as climate change worsens. For example, it is estimated that there will be a nearly 2-fold increase in wildfire-induced summer PM_2.5_ concentrations by 2050 over North America, partially counteracting the improvements from regulations on anthropogenic emissions.^88^ Wildfire PM tends to have a smaller particle size and contains more oxidative and proinflammatory components compared with urban background PM.^87^ Short-term exposure to wildfire PM is associated with nonaccidental, cardiovascular, and respiratory mortality.^89^ In addition, exposure to wildfire smoke may impair lung function and increase the risk for related respiratory events such as hospitalizations, emergency department visits, physician visits, and medication use for asthma, chronic obstructive pulmonary disease, and respiratory infection.^87^ It is currently unclear how such episodic events like wildfires contribute to the findings of long-term PM exposure and health studies. Hence, research on the health effects of prolonged exposure to wildfire PM is a clear research need.

The changing and evolving nature of PM also suggests that extension of the studies in HEI’s low-exposure epidemiology initiative could be informative, say within 5 or 10 years. Relatedly, assessing the health effects of air quality interventions remains of ever-increasing interest,^90,91^ and the HEI studies may be a good avenue for conducting those types of analyses.

### 6.3 The Role of the Indoor Environment

As with most other ambient air pollution and health studies, indoor air pollution was not examined in the current initiative. Outdoor–indoor infiltration rates, time–activity patterns, and indoor sources of air pollution (e.g., cooking) are known to influence total exposure, and most people spend 80% to 90% of their time indoors at homes, schools, and places of work.^92−94^ Lack of consideration of infiltration rates and time-activity adds exposure measurement error, which is often assumed to bias the estimated ambient air pollution and health estimates toward the null, although the nature of the potential bias cannot be fully known (see earlier discussion).

Indoor environments represent a mix of outdoor pollutants that can infiltrate through natural and mechanical ventilation, and contaminants originating inside the building. Indoor contaminant sources include cooking fuels, tobacco and candle combustion, emissions from building materials and furnishings, central heating and cooling systems, humidification devices, moisture processes, electronic equipment, household cleaning products, and pets. Investigating the complex interplay between indoor and ambient air pollution with health is difficult because indoor air pollution is typically not measured for large populations over long periods, and indoor air contains a more diverse range of pollutants than outdoor air.^95^ Such investigation is hampered by the fact that the science on indoor air pollution is relatively underdeveloped with many persisting uncertainties, such as the composition of indoor air pollution, and how pollutants form and accumulate in indoor spaces. Moreover, whereas outdoor-air measurements can be designed to be representative of a wide geographical area, which facilitates large-scale modeling, indoor air quality measurements might relate to only one room given the wide diversity in how buildings are constructed, ventilated, operated, and occupied.^96^ More work is needed on how indoor and outdoor air pollution influence each other. In particular, more data are needed on infiltration factors, how they differ across building types and locations, and how they modify the duration and dose of air pollution exposure of ambient origin. Some of the information on infiltration could be useful in more fully characterizing heterogeneity in risk estimates, including disparities driven by socioeconomic factors. All this information would also be useful to protect health in the largely unregulated indoor environment.

### 6.4 Vulnerable and Susceptible Populations

The HEI low-exposure epidemiology studies focused on mortality outcomes in the general population with national representativeness because those studies are most influential in terms of guiding regulation and associated cost-benefit analyses. Beyond mortality, PM_2.5_ damages most organ systems and is linked to many debilitating diseases.^3,4,6^ Moreover, certain groups are especially vulnerable and more likely to experience adverse health effects of air pollution, including pregnant women, children, the elderly, chronic disease patients, and those of lower SES.^6^ Furthermore, marginalized groups are more likely to live in air pollution hotspot areas, resulting in environmental injustice and additional health disparities.^59,97^

In the United States, it has been well established that low-income communities and communities that are racially segregated and historically marginalized experience a disproportionate health burden from ambient air pollution, and other environmental and social stressors. Those racial-ethnic inequalities in air pollution exposures are attributable in part to structural racism, including historical, race-based housing segregation and land-use practices.^98−100^ The United States has begun to address the challenges of addressing air pollution–health inequalities through the NAAQS process, where additional monitors are required to be placed in marginalized communities.^12^ However, as recent analyses have shown, implementing a (tighter) NAAQS might not be as effective at reducing inequities as targeted location-specific interventions.^101^ Thus, there is a need to enhance policy-relevant research efforts to better address and reduce disproportionate exposures and effects in marginalized communities. Specifically, there is a pressing need to identify and assess which multiple, overlaying (cumulative), chemical and nonchemical stressors lead to environmental health inequities to help focus policies and other actions on the most harmful stressors or combination of stressors. As such, the U.S. EPA and other federal agencies have enhanced their efforts to reduce environmental health inequities under the Justice40 initiative, including support for community-engaged research efforts to study and reduce cumulative environmental impacts.^102,103^ HEI has also recently launched a new environmental justice program to better meet the needs of historically marginalized communities while addressing environmental inequities.^104^

### 6.5 What is the Biological Plausibility of Long-Term Effects at Low Concentrations?

Decades of in vivo and in vitro toxicological studies have addressed the mechanisms by which PM causes adverse health effects.^3,4^ The approaches inevitably involve use of doses that are anticipated to perturb biological systems, with the numbers of particles reaching tissue targets greatly exceeding the cellular doses associated with the exposures investigated in HEI’s low-exposure epidemiology initiative.

Although new epidemiological studies have reported associations with health effects at PM_2.5_ concentrations below current ambient air quality standards in the general population, no human experimental nor toxicological data are currently available that address long-term exposure effects of very low concentrations. We are therefore reliant wholly on analyses of epidemiological data with their attendant uncertainties, although consistency and coherence of the epidemiological evidence are compelling factors in assessing causality. Conceivably, intervention studies and “natural experiments” could be identified and exploited for this purpose.^90,91^

At this time there are no clearly defined mechanisms of action or adverse outcome pathway networks that would explain the multitude of adverse health effects of PM and other air pollutants at very low concentrations. Several mechanisms have been hypothesized for PM_2.5_, such as lung inflammation triggering subsequent systemic inflammation, translocation of PM directly to target organs, and the stimulation of airway irritant receptors with ensuing systemic inflammation and oxidative stress.^67,105^ Arguably only the latter is seemingly a realistic possibility in the very low-concentration context.^106−108^

Marked interindividual variability in the sensitivity of irritant receptors to inhaled triggers could potentially explain the effects at very low concentrations in some (susceptible) individuals. In epidemiology, the lack of evidence for a threshold at low pollution levels has been attributed to large differences in individual sensitivity within the population and the absence of a well-defined threshold within individuals. It should be noted that the apparent lack of a threshold at the population level should not be interpreted as meaning there is no threshold for effects at an individual level.^109^ The level of exposure that can be tolerated without adverse effects (that is, at which physiological responses can be regarded as protective or adaptive, rather than as adverse or of potential clinical relevance) would be expected to vary between individuals. It would also likely vary across the life-course for any given individual, depending upon factors such as age and health status.

Also, hypothesized mechanisms are not mutually exclusive and different mechanisms may be in play at different exposure levels, raising the notion of dose-dependent transitions.^110^ For example, one mechanism could be involved predominantly at very low concentrations, which then becomes saturated, leading to involvement of other mechanisms that were not in play at low concentrations. So, while PM effects at very low concentrations are not implausible, at this time, these possibilities are largely speculative and await further progress in understanding the mechanisms of air pollution effects.

## Conclusion

In conclusion, HEI’s low-exposure epidemiology initiative contributed to the growing body of epidemiological evidence regarding associations between air pollution and health at today’s low levels of ambient air pollution in North America and parts of Europe.

All three studies documented positive associations between mortality and PM_2.5_ at concentrations below the U.S. NAAQS and current and proposed European Union limit values. Furthermore, the studies observed linear or supra-linear ERFs between PM_2.5_ and mortality, with no evidence of a threshold. Substantial heterogeneity was found both in the magnitude— not direction—and shape of the PM_2.5_ association within and across studies. This heterogeneity may be informative and warrants further examination. Overall, evidence from those studies provides additional support for the 2021 WHO AQG for annual PM_2.5_ of 5 μg/m^3^ and NO_2_ of 10 μg/m^3^.

This research initiative provided important new evidence of the adverse effects of long-term exposures to low levels of air pollution at and below current standards, suggesting that further reductions could yield larger benefits than previously anticipated.
